# Ionic Liquids as Reconditioning Agents for Paper Artifacts

**DOI:** 10.3390/molecules29050963

**Published:** 2024-02-22

**Authors:** Catalin Croitoru, Ionut Claudiu Roata

**Affiliations:** Materials Engineering and Welding Department, Transilvania University of Brasov, Eroilor 29 Str., 500039 Brasov, Romania; ionut.roata@unitbv.ro

**Keywords:** paper artifacts, cellulose, ionic liquids, cleaning agents, preservation

## Abstract

This research explores the potential of ionic liquids (ILs) in restoring paper artifacts, particularly an aged book sample. Three distinct ILs—1-ethyl-3-propylimidazolium bis(trifluoromethylsulfonyl)imide, 1-methyl-3-pentylimidazolium bis(trifluoromethylsulfonyl)imide, and 1-methyl-3-heptylimidazolium bis(trifluoromethylsulfonyl)imide —both in their pure form and isopropanol mixtures, were examined for their specific consumption in conjunction with paper, with 1-ethyl-3-propylimidazolium bis(trifluoromethylsulfonyl)imide displaying the highest absorption. Notably, the methyl-3-heptylimidazolium ionic liquid displayed pronounced deacidification capabilities, elevating the paper pH close to a neutral 7. The treated paper exhibited significant color enhancements, particularly with 1-heptyl-3-methylimidazolium and 1-pentyl-3-methylimidazolium ILs, as evidenced by CIE-Lab* parameters. An exploration of ILs as potential UV stabilizers for paper unveiled promising outcomes, with 1-heptyl-3-methylimidazolium IL demonstrating minimal yellowing post-UV irradiation. FTIR spectra elucidated structural alterations, underscoring the efficacy of ILs in removing small-molecular additives and macromolecules. The study also addressed the preservation of inked artifacts during cleaning, showcasing ILs’ ability to solubilize iron gall ink, particularly the one with the 1-ethyl-3-propylimidazolium cation. While exercising caution for prolonged use on inked supports is still recommended, ILs are shown here to be valuable for cleaning ink-stained surfaces, establishing their effectiveness in paper restoration and cultural heritage preservation.

## 1. Introduction

Preserving and restoring cellulose paper artifacts, such as books, is of utmost importance in order to protect our cultural heritage and ensure their longevity [1,2,3]. However, over time, cellulose artifacts are subject to various processes that can lead to degradation [4,5]. Understanding degradation and implementing effective restoration methods is crucial for the preservation of these materials [6]. The altering of cellulose paper involves intricate mechanisms, often stemming from a combination of factors such as exposure to microorganisms, light, humidity, temperature fluctuations, and pollutants [7,8,9]. Endogenous factors, such as the inherent quality of cellulose fibers, the presence of lignin, metallic ions, and various compounds used as sizers and binders, further contribute to the degradation process [5,10,11]. These factors collectively lead to physical damage, discoloration, staining, and acidification, ultimately compromising the structural integrity of the paper [10].

Traditional methods for restoration involve color restoration (including the removal of various types of stains), and deacidification. Color restoration aims to revive the original vibrancy of the paper, while deacidification helps neutralize the acidity that can accelerate degradation [12,13,14].

Color restoration and cleaning techniques can be time-consuming multi-step processes and may involve the use of chemicals such as hydrogen peroxide, chlorine or hypochlorites [15], enzymes [16] or even gamma radiation [17] and laser beams [18] that can be harsh on the paper leading to alterations in its mechanical properties and original appearance. Additionally, attempts to use mechanical cleaning methods, such as erasing and abrasive cleaning, may result in damage to the paper [19]. Post-production deacidification methods, such as non-aqueous solvent cleaning or spraying with alkaline preserves, may not always effectively remove all acidity or can cause structural damage to cellulose in the long term [14,20,21].

Irrespective of the chosen restoration methods and techniques, they must adhere to fundamental criteria, with paramount importance given to increased efficiency and minimal potential for paper substrate degradation [3,22]. Preserving the original appearance and texture of the material, ensuring long-term stability for any introduced additives or consolidants, and maintaining environmental friendliness with low toxicity are equally pivotal considerations [23]. These criteria collectively form the essential framework for evaluating the efficacy and sustainability of any restoration approach, emphasizing the need for holistic and conscientious practices in the preservation of cultural heritage materials. A critical analysis of the restoration and conservation methods to date for cellulosic materials shows that all traditional methods basically cannot meet all these criteria simultaneously [23,24]. Although these traditional methods have proven effective in a strict sense, such as cost efficiency or end result, they certainly have drawbacks with regard to other aspects.

These challenges highlight the need for alternative restoration methods that can address these limitations and provide a more effective, gentle, and sustainable approach to preserving cultural heritage [25].

One potential solution that has emerged in recent years is the use of ionic liquids (ILs) in conjunction with artifact preservation [25,26,27]. Ionic liquids are composed entirely of ions, which gives them unique properties that make them suitable for restoration purposes. Their cleaning and color restoration effect is based on the ability to partly dissolve cellulose and other macro- or small-molecular compounds allowing for the removal of stains and contaminants [28,29,30]. They can also be used to impart antibacterial or antifungal character to the paper surface for improved preservation by disrupting the cell wall membranes or by interfering with the metabolism of degrading microorganisms [23,31]. In addition to these properties, they also present low vapor pressure, high chemical stability, tunability and recyclability, which allows for the optimization and reuse of IL-based reconditioning formulations. However, ILs can also dissolve and damage cellulose, the main component of paper, which can result in the loss of mechanical and structural integrity of paper [31,32]. Therefore, there is a tradeoff between the cleaning and solvency effects of ILs, which depends on the type and concentration of ILs, the duration and temperature of the treatment, and the composition and condition of the paper. To minimize the solvency effect of ILs on cellulose and maximize the cleaning effect of ILs on paper, it is preferable to use ILs with longer alkyl chain cations or ILs with anions that do not have a strong impact on cellulose [32].

There are limited studies in the literature on the direct application of ILs on old and degraded book cellulose substrates. Most of the information available is indirect, and is on their application as wood preservatives, in cellulose dissolution and recovery, as topical biocides, or as solvents for various systems [23,26,33]. Although their long-term impact on artifacts is not yet completely understood, the specific use of ionic liquids in cellulose preservation has shown promising results. Ionic liquids have the ability to stabilize the pH of the paper, preventing further degradation [23,30]. Ionic liquids with short side alkyl chains such as 1-butyl-3-methylimidazolium benzotriazole and 1-butyl-3-methylimidazolium 1,2,4-triazolate showed the highest deacidification capability, increasing the paper pH to more than 7 with solutions at concentrations above 3%, but caused modifications in paper texture and transparency [23].

Also, due to their plasticizing effect on cellulose and lignin, ILs can improve the mechanical resistance of paper artifacts by increasing their tensile strength, fracture toughness, elongation, burst index, tear index, and fold endurance [34,35,36].

Ionic liquids, particularly protic ionic liquids (PILs) and those with azole-functionalities, have been found to exhibit significant biocidal and antibacterial properties when used in the restoration of cellulose paper in artifacts. PILs with 1-ammonium-2-propanol cation and various anions have shown antimicrobial activity against a range of microorganisms, including fungi, bacteria, and yeasts [37]. Furthermore, the addition of specific ionic liquids, such as benzalkonium nitrate(V), benzalkonium DL-lactate, and didecylodimethylammonium DL-lactate, to paper samples or pine bleached kraft pulp has been shown effective against paper-infesting molds and yeasts [23,27,31].

In this study, we explore novel approaches to preserving and restoring cellulose paper artifacts, with a specific focus on books, aiming to overcome the challenges posed by their inevitable degradation over time. The study utilizes three ionic liquids, specifically 1-ethyl-3-propylimidazolium bis(trifluoromethylsulfonyl)imide (PrEIMTs), 1-methyl-3-pentylimidazolium bis(trifluoromethylsulfonyl)imide (PentMIMTs), and 1-heptyl-3-methylimidazolium bis(trifluoromethylsulfonyl)imide (HeptMIMTs). By subjecting paper strips from an aged book to short-term immersion treatments in IL/isopropyl alcohol mixtures, the study systematically assesses the impact of these treatments on the paper color restoration, stain removal and deacidification. The chosen ILs are able to clean the paper supports and practically completely deacidify them in much shorter time periods (6 h) than those reported in the literature (typically over 24 h), without modification in the paper’s texture or opacity. Additionally, the research investigates the potential of ILs to act as UV stabilizers, offering protection against UV-induced color alterations, a novel application not extensively explored in previous literature. Moreover, the study delves into the interaction between ILs and iron gall ink, a common ink associated with paper artifacts [38], demonstrating the compatibility of IL treatment with inked paper supports, which is crucial for preserving handwritten or printed content during restoration processes. Overall, by addressing these aspects, this study contributes to the advancement of restoration techniques for cellulose paper artifacts, offering a more effective, gentle, and sustainable approach to preserving cultural heritage materials.

## 2. Results and Discussion

**Figure 1:**
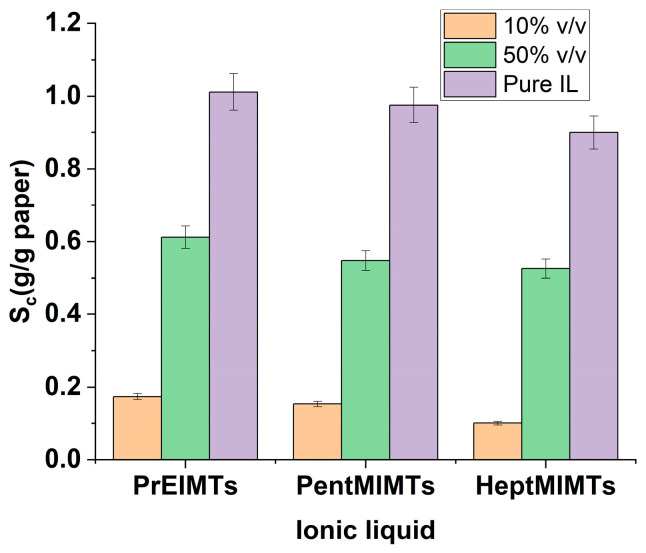
The specific consumption (S_c_) of pure ILs and ILs/isopropanol mixtures for Whatman filter paper.

The specific consumption (S_c_) values of ionic liquids from both mixtures with isopropanol (10% *v*/*v* and 50% *v*/*v*), as well as those of pure ILs, for Whatman filter paper, are depicted in Figure 1. Typically, S_c_ increases with the IL amount and is also influenced by the molar mass of the IL. Consequently, the PrEIMTs ionic liquid is absorbed in the highest amount.

This absorption trend remains consistent, and the same pattern is evident for the B1 paper sample, particularly in the case of the 10% *v*/*v* mixture with isopropanol, as shown in Table 1. It is worth noting that the values are slightly lower, attributable to the higher compactness of the book paper. Information on the specific consumption of ILs for paper treatment in the reference literature is limited and inconsistently reported. For the sake of comparison, it is noted that specific consumption values for 1-butyl-3-methylimidazolium ILs with triazole anions were within the range of 0.5 to 2 g/g of paper, but these values were obtained with a 24 h treatment duration [23].

The treatment of the B1 paper revealed that the ionic liquid HeptMIMTs exhibited a good capability for deacidification of paper. The 10% solution of this IL in isopropanol markedly increased the pH to 7.00, compared to the initial pH of 4.30. The other ILs caused a marginally lower shift in pH, but they could be deemed efficient for this purpose as well. Similar effective deacidification of old paper has been reported for small-chain alkylimidazolium halides but for a longer treatment duration [23].

There are several mechanisms that can cause paper acidification, such as the partial degradation or hydrolysis of acidic raw materials used during the papermaking process, which degrade over time and form organic acids, such as acetic, formic, or oxalic acids, or inorganic acids, such as sulfuric or aluminum sulfate acids, that can lower the pH of paper. Another mechanism involves acid hydrolysis that occurs when paper is exposed to moisture and heat, which breaks the bonds between the glucose units of cellulose, the main component of paper, and releases protons. The protons then react with the remaining cellulose chains, forming more water and reducing the pH of paper.

Ionic liquids can deacidify paper by dissolving and extracting various organic and inorganic compounds from paper, such as lignin, hemicellulose, cellulose, metal ions, and salts, which can reduce the amount and therefore effect of acidic species in the paper and increase its pH [39]. This is the main mechanism of paper deacidification by Ils, as evidenced by the FTIR studies (for organic species) and by the lower ash content of the supports treated with Ils compared to B1 (for inorganic species) (Table 1). Also, there seems to be a slight reduction in the paper’s basis weight of the treated supports compared to B1, which could further sustain the washing effect of the ILs on paper. However, other mechanisms may also contribute to the deacidification effect, such as the hydrogen bonding between ILs and cellulose, which may alter the acidity and basicity of the hydroxyl groups, and the other types of interactions between ILs and paper, such as electrostatic, van der Waals, or π–π stacking interactions, which may influence the solubility and stability of paper components [36,40]. The ILs used in this study have the same anion, and similar cations, which are N-alkyl-N′-methylimidazolium derivatives with different alkyl chain lengths. The sulfonylimide anion is a weak base, with a pKa of about 5.5, and it is unlikely to accept a proton from a weak acid, such as the carboxylic acids that could be present in the paper [41]. Moreover, the imide anion is highly polarizable and delocalized, which reduces its basicity and increases its interaction with the ILs cation. Therefore, the reaction between the imide anion and the protons in the paper is probably negligible or insignificant. However, the cation and the alkyl chain length may play a role in the interaction with paper, as they affect the solvation, dispersion, and penetration of the ILs into the paper structure.

**Figure 2:**
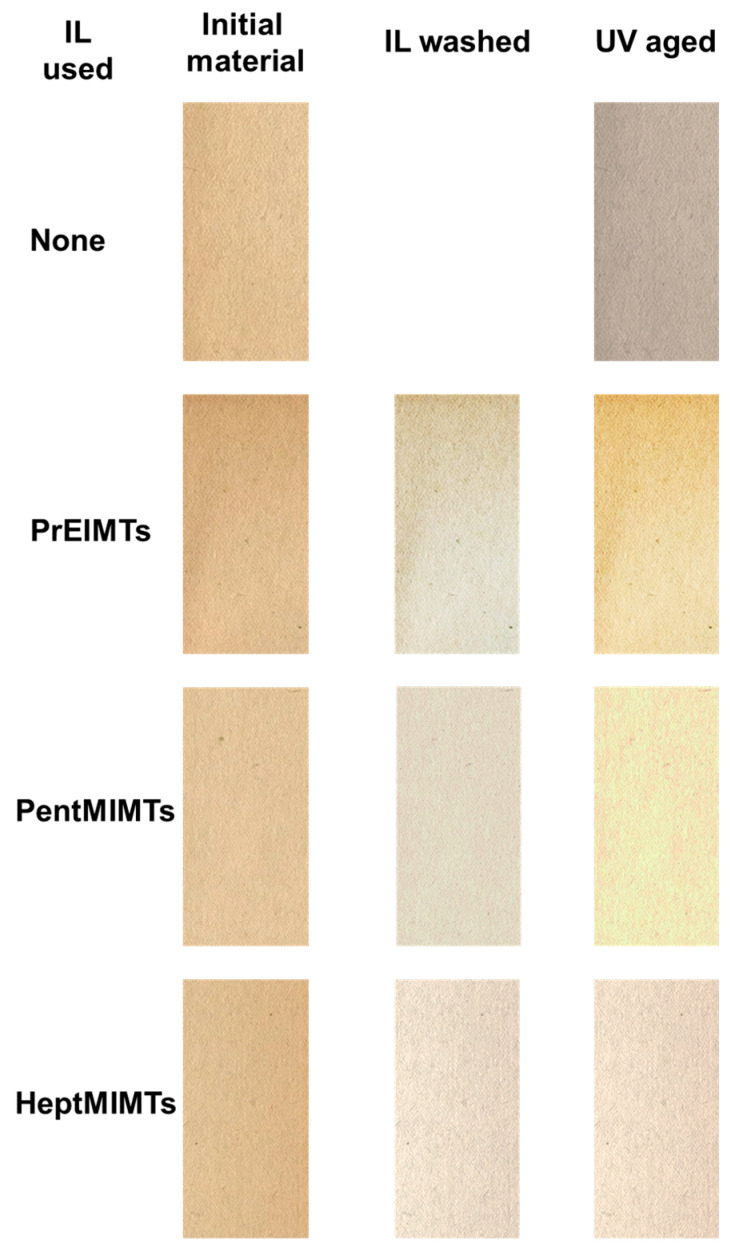
Photographic images of the initial B1 paper strips, of the same strips cleaned with IL/isopropanol solutions and IL-treated strips submitted to accelerated UVA aging (254 nm).

**Figure 3:**
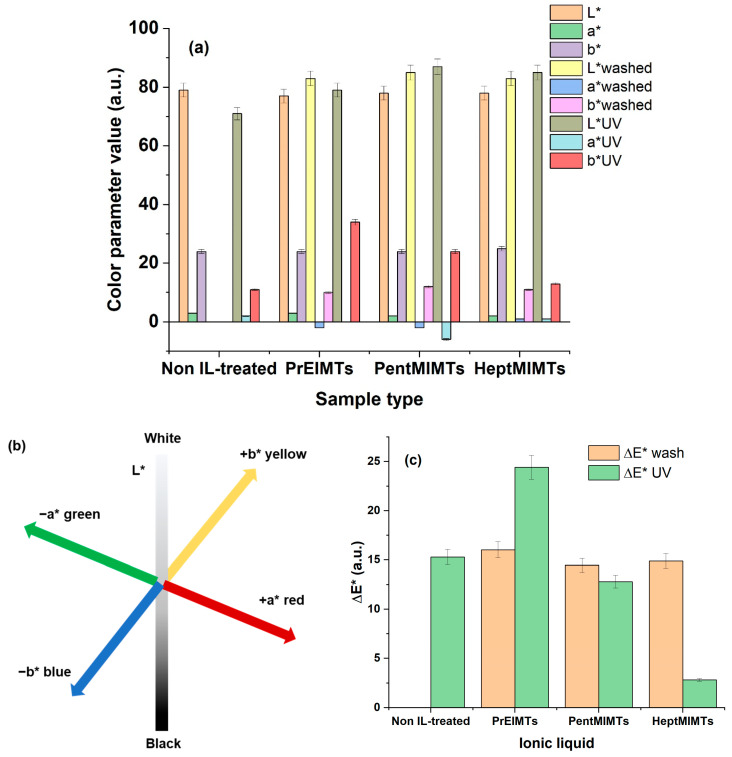
(**a**) CIE-Lab* color parameters for the IL-treated paper samples and for the samples submitted to UV aging; (**b**) CIE-Lab* space color coordinates; (**c**) total color modifications for the washing step and accelerated UV aging.

Figure 2 illustrates the modifications brought to the paper by the IL-cleaning process studied here, while Figure 3a–c provide the quantitative CIE-Lab* parameters to assess those modifications and their interpretation.

The B1 paper material exhibits a noticeable yellow/reddish hue, as evidenced by positive values in the a* and b* parameters (Figure 3a,b). The immersion of the paper in 10% IL solutions in isopropanol effectively “washes” the paper, restoring whiteness (by up to 6%) and eliminating a portion of the respective tinges that contribute to the paper’s aged/degraded appearance (a decrease of up to 20% in a* values and 55% in b* values, as shown in Figure 3a). Notably, the highest color differences are observed for HeptMIMTs and PentMIMTs ILs, in Figure 3c.

To assess as much as possible all the benefits of the IL treatment of aged paper, the possibility of extending the applicability of these compounds as UV stabilizers for this type of material was studied. The effect of color changes on IL-treated paper samples and on B1 reference under UVA irradiation aging is shown in Figure 2 and Figure 3a–c. While irradiation could drastically affect the color of the untreated material, the modifications in color of the IL-treated samples are less pronounced. With irradiation, the color of the B1 paper samples became significantly darker (a decrease in the lightness parameter, L*) and bluish/greenish, consistent with other findings [42,43].

The UV-aged IL-treated paper strips actually presented a minimal increase in lightness (under 5%), probably due to the ILs inducing glossiness to the surface [43] and restructuring the material, coupled with a yellowing/reddening of the material. For the sample treated with HeptMIMTs, only a 1% in yellowing and 2% total color modifications were observed from the b* values and ΔE*, respectively, possibly indicating that this IL would be best suited for the UV stabilizing of paper. This is consistent with our previous studies on wood and cellulose fibers, where the IL with the highest lateral alkyl chain also proved to be the most effective in inhibiting the formation of free radicals and chromophore groups responsible for yellowing/reddening [34,44,45].

**Figure 4:**
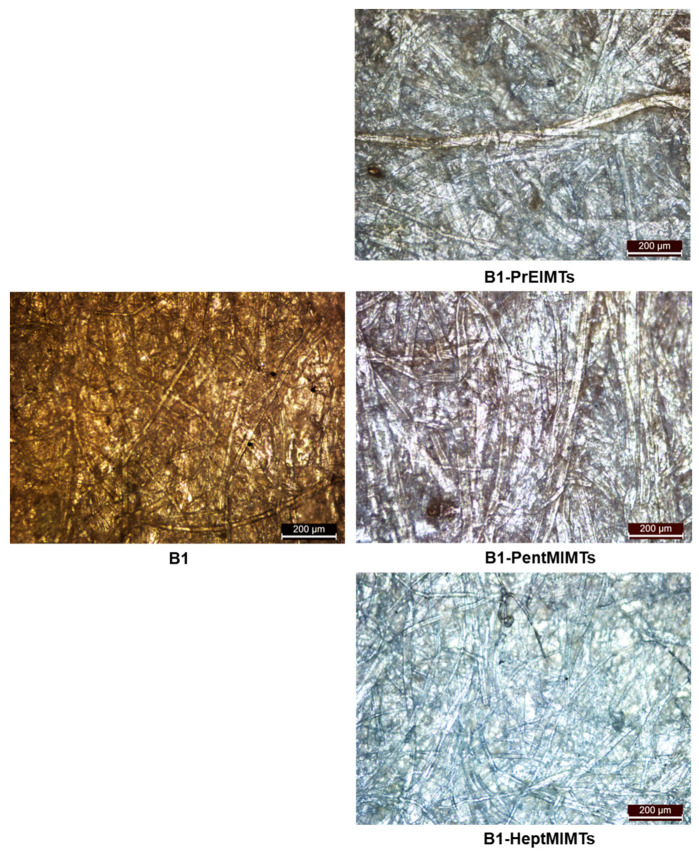
Optical microscopy images of paper strips used in the washing treatment of B1 paper substrates with IL 10% *v*/*v* solutions (100×).

As evidenced from the optical microscopy images (Figure 4), the fibers in the untreated paper (B1) appear compact and closely knit (probably due to the binder), indicating that they have a small diameter and a high density. The fibers in the paper treated with PrEIMTs (B1-PrEIMTs) appear slightly more separated and cleaner than B1, suggesting that the ionic liquid has dissolved and removed some of the stains, dirt, and degraded compounds from the paper surface. Also, this ionic liquid has caused swelling of the fibers, increased their diameter and reduced their density to the highest extent.

The fibers in the paper treated with PentMIMTs (B1-PentMIMTs) show more distinct and visible fibers than B1-PrEIMTs, indicating that the ionic liquid has a stronger cleaning effect than PrEIMTs, but with some variation or inconsistency.

The fibers in the paper treated with HeptMIMTs (B1-HeptMIMTs) also show distinct fibers, but with less clarity than B1-PentMIMTs. This may suggest that the ionic liquid has a similar cleaning or swelling effect as PentMIMTs. The fibers have a comparable diameter and density as B1-PentMIMTs, and the red-brownish grime was mobilized to the highest extent.

This variation in the cleaning effect of the ILs suggest that their efficiency in this case is not only influenced by their solvency effect, but other factors may play a role as well, such as their surface tension, as higher molar mass ILs are known to possess a surfactant character [46].

The swelling effect is the expansion of the cellulose fibers due to the penetration of the IL molecules into the fiber structure. The average cellulose fiber diameters increased after the IL treatment, in the order: B1 (12.5 µm) < B1-HeptMIMTs (14.3 µm) < B1-PentMIMTs (15.4 µm) < B1-PrEIMTs (17.5 µm). This order corresponds to the decreasing crystallinity index and the increasing hydrogen bond disruption ability of the ILs, as confirmed by the FTIR spectra analysis. The increase in diameter due to fiber swelling ranged from 13% for HeptMIMTs to 18% for PentIMIMTs. This increase is typical also for the paper industry (pulp refining increases the initial fiber diameters by 5–10%) [47], as well as for the domain of paper restoration. For example, the fiber diameter can increase by 10–20% after alkaline treatment, depending on the concentration and duration of the treatment [48].

**Figure 5:**
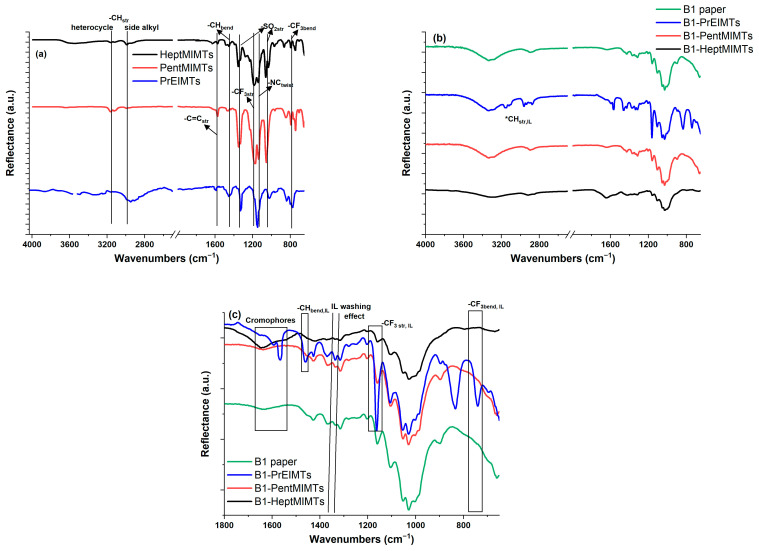
FTIR spectra of (**a**) ILs; (**b**) B1 paper strip and B1 strips treated with ILs; (**c**) zoom in the fingerprint region of B1 paper strip and B1 strips treated with ILs.

The distinctive features of the IL washing agents in their FTIR spectra from Figure 5a manifest primarily in two regions: the CH stretching region (2800–3180 cm^−1^) and the bond-stretching/fingerprint region (below 1800 cm^−1^). Most notably, in the -CH stretching region, the band at 3150–3170 cm^−1^ corresponds to the symmetric stretching of H-C-C-H in the imidazolium ring, along with stretching of the N(CH)N C-H ring [49,50]. Within the bond-stretching/fingerprint region, notable bands for the cation include those at ~1565 cm^−1^ (C=C stretch), at ~1132 cm^−1^ (-N-CH_3_ twisting) and several weak overtones below 800 cm^−1^ [51]. The Ts anion contributes strong bands at ~785 cm^−1^ and 1185 cm^−1^, associated with CF_3_ symmetric bending and stretching. Other bands at 1045 cm^−1^ and 1335 cm^−1^ indicate SO_2_ stretching, complemented by several overtones of medium intensity below 800 cm^−1^ [52]. The lowest molar mass IL, PrEIMTs, exhibits traces of adsorbed water, evidenced by -OH stretching vibrations at ~3360 cm^−1^.

Following treatment with the three ionic liquids (ILs), the respective FTIR spectra of B1-PrEIMTs, B1-PentMIMTs, and B1-HeptMIMTs samples exhibit distinctive bands associated with both the IL cation (Figure 5b, imidazolium -CH stretch) and, more prominently, the anion (Figure 4c, -CF_3_ stretch modes).

To evaluate the structural changes in the paper supports, the crystallinity index for cellulose was estimated by calculating the hydrogen bond index (HBI), which is a ratio of the intensity of the O-H stretching band at 3315 cm^−1^ to the intensity of the -CH and -CH_2_ bending band at 1315 cm^−1^. The HBI reflects the degree of hydrogen bonding between the hydroxyl groups of cellulose, which is lower in the crystalline region and higher in the amorphous region. Therefore, the higher the HBI, the lower the crystallinity index, as more hydroxyl groups are involved in hydrogen bonding in the amorphous region, which results from the transformation of cellulose I (crystalline) to cellulose II (amorphous) [53].

The IL treatment of the B1 sample at the relatively low concentration of 10% *v*/*v* in this study appears to have a moderate impact on the material’s structure. No additional bands, beyond those associated with the main components in paper and the IL, were identified. The overall shape of the spectra and the relative ratio between the main bands attributed to the cellulose structural backbone, such as the -C-O-C- α-1,4 glycosidic linkages (1149 cm^−1^) and the glycosidic ring (1078 cm^−1^) [43], appear largely unchanged.

The HBI values decrease in the order B1-PrMIMTs (4.68) > B1-PentMIMTs (4.20) > B1 (4.00) > B1-HeptMIMTs (3.95), indicating that the lower mass IL (PrMIMTs) had the highest cellulose hydrogen bond disruption potential.

There are clear indications that the treatment promotes the removal of small-molecular (partly degraded) paper additives, macromolecules (cellulose, lignin) and acidic species due to the effective solvation ability of the ILs. For example, in the chromophore group region highlighted in Figure 5c, the aged paper exhibits weak stretching vibration modes specific to conjugated C=O in xylans (1582 cm^−1^) and C=C stretching in lignin (1600 cm^−1^) [54,55]. Conversely, an increase in the intensity of bands associated with lignin and xylan was observed for B1-PrEIMTs, B1-PentMIMTs, and B1-HeptMIMTs, likely due to the ILs’ structure-mobilizing effect. The ILs seem to facilitate the removal of partly degraded macromolecules contributing to paper yellowing, while the restructuring of cellulose exposes a higher number of bonds belonging to both lignin and xylan units to the IR beam, maintaining or even enhancing the overall band intensity.

The FTIR spectra in Figure 5c also provide insights into the nature of paper additives. The broad absorption at ~1635 cm^−1^ in B1 could be attributed to amide I bands, potentially from gelatin [56,57], while weak bands at 1357 and 1329 cm^−1^ may be associated with fatty acid salts like stearates (commonly used as paper additives) or even lipids characteristic of molds or fungi [58]. All three ILs appear to mobilize these small-molecular compounds, known to accumulate dirt and contribute to yellowing over time, leading to the partial restoration of paper color, as observed in Figure 2 and Figure 3.

Since cellulosic paper artifacts that need cleaning and restoration are usually written, drawn or printed on, in conjunction with their cleaning effect, other important aspects of ILs are related to their maintaining intact the respective addition drawing/text during the cleaning process and that the ILs mobilize preferentially only the degraded compounds, not the inks, pigments or toners.

**Figure 6:**
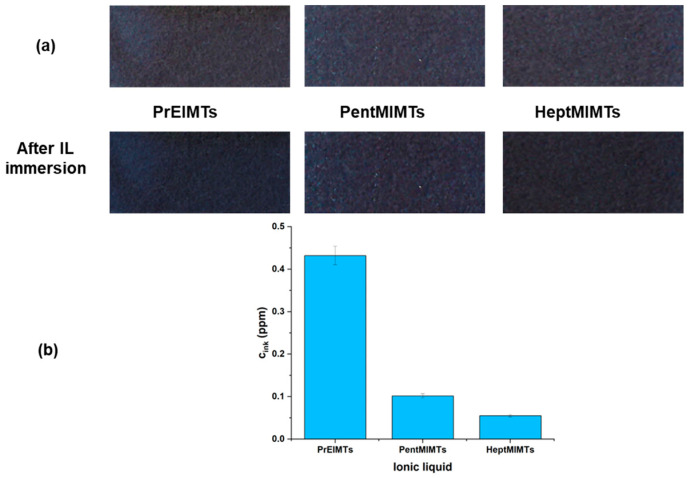
(**a**) Photographic images of the dyed Whatman paper strips immersed in ILs; (**b**) concentration of leached ink in the ILs.

The spectrophotometric VIS analysis of the pure ILs in which the ink-dyed Whatman filter paper strips were immersed for 24 h (Figure 6) have shown that all of the ILs have the ability to solubilize a low amount of the iron gall ink. Even if this leached amount is very low and could actually represent the excess of sorbed ink on the surface of the paper, while visually the strips remain basically unchanged, the exact impact on a real-life artifact needs to be further assessed.

The highest solvation ability for the iron gall ink is attributed to the lowest molar mass IL—PrEIMTs—while for the longer alkyl-chain ones, the leached amounts are close to the detection limit of the instrument. To evaluate the compatibility of the ILs with handwritten supports, a strip of B1 paper was inscribed with various lines and word-like shapes using iron gall ink and a fountain pen with a O type calligraphy nib. After drying for 24 h, the strip was immersed in the HeptMIMTs 10% *v*/*v* isopropanol solution for 6 h and then extracted, dried and visually inspected. HeptMIMTs is an IL that has a long alkyl chain cation and a weakly basic anion, which can reduce the solvency and reactivity of the IL towards cellulose and iron gall ink, respectively.

**Figure 7:**
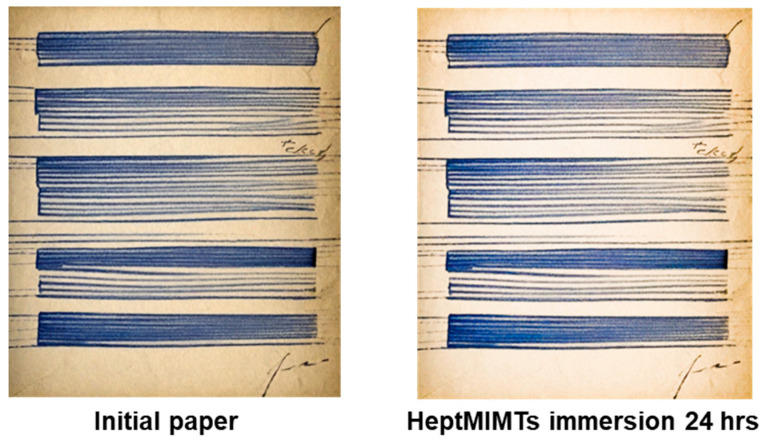
Photographic images of the inked B1 paper supports before and after 6 h immersion in the HeptMIMTs 10% *v*/*v* isopropanol solution.

Figure 7 shows that the iron gall ink drawings remained intact and unaffected after the IL treatment, indicating that HeptMIMTs did not compromise or erase the integrity of the ink, while the paper itself became lighter and cleaner after the IL treatment.

## 3. Materials and Methods

The ionic liquids employed in this study—1-ethyl-3-propylimidazolium bis(trifluoromethylsulfonyl)imide (C_10_H_15_F_6_N_3_O_4_S_2_), 1-methyl-3-pentylimidazolium bis(trifluoromethylsulfonyl)imide (C_11_H_17_F_6_N_3_O_4_S_2_), and 1-heptyl-3-methylimidazolium bis(trifluoromethylsulfonyl)imide (C_13_H_21_F_6_N_3_O_4_S_2_)—were sourced from IoLiTec Ionic Liquids Technologies GmbH, Heilbronn, Germany, with a purity of 98% or greater (Table 2). Isopropyl alcohol (Sigma-Aldrich, Darmstadt, Germany, >99% vol.) was used as a basis to prepare 10% *v*/*v* and 50% *v*/*v* mixtures with the three ILs.

Paper strips were extracted from a book published in 1938 (B1). Several characteristics of this paper, such as its basis weight, pH and basic composition are given in Table 2. A Whatman qualitative filter Grade 1, with α-cellulose content surpassing 98%, served as a reference for comparative analysis.

Before immersion in the reconditioning solutions and pure ionic liquids, all the paper strips underwent a conditioning period of 24 h at 23 °C ± 1 °C and 50 ± 2% relative humidity within a desiccator containing a supersaturated Mg(NO_3_)_2_ solution. The initial weight of the paper strips was measured (m_0_) to establish a baseline for comparative evaluations.

The Whatman filter paper strips underwent immersion in pure ionic liquids (ILs) as well as in 10% *v*/*v* and 50% *v*/*v* IL/isopropyl alcohol mixtures. The strips were periodically removed and weighed until reaching a state of constant mass, indicating equilibrium sorption. This equilibration process took approximately 6 h from the start of immersion. Subsequent to achieving equilibrium, the samples underwent conditioning by following the previously outlined procedure and were reweighed (m_f_).

Since isopropyl alcohol is volatile enough to undergo complete evaporation, the paper strips in this scenario retained only the ionic liquids. The specific consumption of IL, reported per gram of paper (S_c_), was calculated using Equation (1) [23]:

(1) $$S_{c}=\frac{m_{f}-m_{0}}{m_{0}}$$

For the treatment of B1 paper strips, a 10% *v*/*v* solution of ionic liquids (ILs) in isopropyl alcohol was specifically selected, coupled with a 6 h treatment duration. This choice was made to facilitate the rapid absorption of the solution into the paper strips, owing to its lower viscosity and the minimal IL content.

The ILs used in this study have the same anion—bis(trifluoromethylsulfonyl)imide—which can form weak hydrogen bonds with cellulose and small-molecular compounds [59,60,61]. They also have similar cations, which are N-alkyl-N′-methylimidazolium derivatives with different alkyl chain lengths. These cations have acidic protons on the imidazolium ring that can form C-H⋯O hydrogen bonds with the compounds found in paper, which partly explains their cleaning effect and deacidification capabilities. However, the cations also have side alkyl chains that can reduce the solubility of cellulose by steric hindrance or hydrophobic interactions [60], limiting potential damage in uncontrolled swelling or dissolution of cellulose fibers. Therefore, the interaction of these ILs with cellulose depend on the balance between the anion and the cation effects, as well as the length of the alkyl chain. The ILs from this study were chosen because in our preliminary screening studies they did not impart modifications to paper texture, in contrast to short alkyl chain ILs, such as 1-ethyl-3-methylimidazolium or 1-butyl-3-methylimidazolium with chloride or acetate anions, reported in the literature as solvents for cellulose [28].

To safeguard valuable cellulosic artifacts, extend their longevity, and mitigate potential long-term damage—coupled with considerations of economic feasibility—the decision was made to exclusively employ the 10% *v*/*v* concentration in the treatment of B1 paper samples.

**Table 2:** Characteristics of the used ILs and B1 paper.

Ionic Liquids Used ^1^	B1 Paper Characteristics
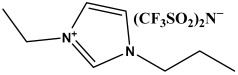 PrEIMTs (391)	pH ^2^: 4.30 ± 0.15
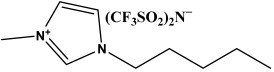 PentMIMTs (405)	Paper basis weight: 67 g/m^2^
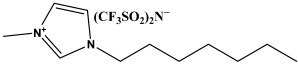 HeptMIMTs (433)	Composition ^3^: 57% groundwood fiber content, 43% bleached chemical pulp, 1.1% ash content

^1^ Molar masses of ILs are given in parenthesis; ^2^ the pH values of paper were determined in accordance with Test Method TAPPI/ANSI T 529 om-21 [62]; ^3^ determined according to [63] (fiber and chemical pulp content) and [64] (ash content), average of triplicate measurements.

The B1 paper strips underwent an identical treatment and conditioning process as the Whatman filter paper. Subsequent to the treatment, the pH of the paper was measured following the T 529-om-21 protocol [62]. This measurement served to assess the efficacy of ionic liquids (ILs) in deacidifying the paper.

The IL-treated paper samples were coded as B1-PrEIMTs, B1-PentMIMTs, and B1-HeptMIMTs throughout this work.

To evaluate the potential advantages of ionic liquids (ILs) in the cleaning process of paper, color modifications in the B1 paper substrates before and after IL treatment were analyzed using a PCEXXM30 colorimeter (PCE Instruments, Meschede, Germany), operating within the CIE-Lab* color space. In this color space, L* signifies lightness, while a* and b* denote the red/green and yellow/blue coordinates, respectively. The IL-treated B1 strips underwent an additional accelerated UV weathering test at 23 °C and 54% relative humidity within a Bio-Link model BLX-E254 (Fisher Scientific, Wien, Austria) irradiation chamber, exposed to 254 nm wavelength for 10 h at a total energy density of 8 mJ/cm^2^. Post-irradiation, color parameters were re-measured to assess any changes and gauge the potential efficacy of ILs as UV stabilizers. This approach aimed to quantify both the immediate impact of IL treatment on paper color and its potential role in providing protection against UV-induced color alterations.

To calculate the total color differences (ΔE*) for each sample, the following equation (Equation (2)) was utilized [65]. The subscripts “*f*” and “*i*” correspond to the values of the color parameters after UV aging and IL treatment, and before, respectively:

(2) $$\Delta E^{*}=\sqrt{(L_{f}^{*}-L_{i}^{*}{)}^{2}+(a_{f}^{*}-a_{i}^{*}{)}^{2}+(b_{f}^{*}-b_{i}^{*}{)}^{2}}$$

The optical microscopy images were acquired with a Leica DM_ILM microscope (Leica Microsystems, Wetzlar, Germany).

ATR-FTIR spectra of the ILs, B1 paper and IL-treated B1 paper were acquired using a Perkin-Elmer Spectrum BXII spectrometer (Waltham, MA, USA) in the spectral range of 650–4000 cm^−1^. The spectra were obtained with a scan step of 2 cm^−1^, and 10 averaged scans were recorded for each sample.

The impact of ionic liquids on iron gall ink, a specific ink commonly associated with paper artifacts, was investigated in this study. Whatman filter paper strips were immersed for 15 min in an iron gall ink formulation (KWZ Iron Gall Ink, Blue-Black, Archive Iron Gall type, Warsaw, Poland). Subsequently, the dyed paper strips were clipped on string supports and allowed to vertically air-dry for 24 h before initiating ink stability testing.

**Figure 8:**
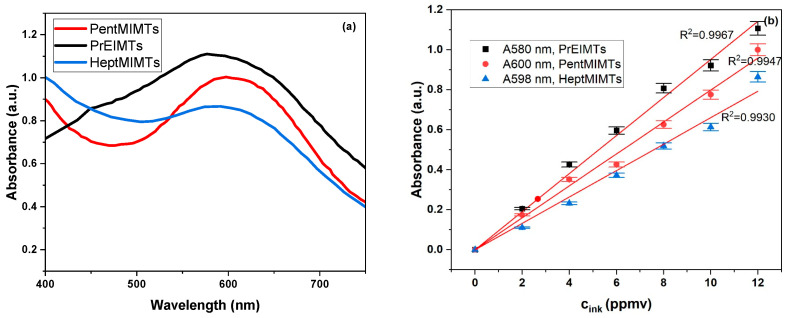
(**a**) VIS spectra of the iron gall ink in the three ILs at 12 ppm concentration; (**b**) the calibration curves for the ink dissolved in the three ILs.

For the evaluation of ink stability, the dyed paper strips were immersed in pure ionic liquids for 24 h at room temperature (22 °C). Following this, the ionic liquids underwent spectrophotometric analysis to ascertain whether the ink leached from the paper supports. The spectrophotometric analysis, conducted on a UV-VIS TU1801 spectrophotometer (DIYpower Co., Ltd., Guangzhou, China) within the range of 400 to 750 nm, utilized the neat ionic liquid as a background reference. Several ink solutions in ILs were prepared, in concentrations ranging from 2 to 12 ppm. Notably, Figure 8a (for the maximum 12 ppm concentration) illustrates that the ink exhibits a broad absorption maximum, dependent on the type of ionic liquid, spanning from 565 nm (for PrEIMTs) to 600 nm for PentMIMTs.

Calibration curves, depicted in Figure 8b, were constructed for the ink dissolved in each respective ionic liquid in a concentration range from 2 to 12 ppm. These curves served as a quantitative tool to evaluate the extent of paper de-inking achieved by each IL. The ILs in which the dyed Whatman paper strips had been immersed underwent subsequent spectrophotometric analysis with neat ILs as background, to quantitatively assess the de-inking of the paper supports.

## 4. Conclusions

In conclusion, this study underscores the significant potential of ionic liquids (ILs) in the restoration of aged paper artifacts, with specific findings highlighting the prominent deacidification capabilities of ILs with the 1-heptyl-3-methylimidazolium cation, leading to a neutral pH. The observed color enhancements, particularly with 1-heptyl-3-methylimidazolium and 1-pentyl-3-methylimidazolium ILs, present promising prospects for revitalizing aged or degraded paper. Furthermore, the investigation into ILs as UV stabilizers for paper reveals encouraging outcomes, emphasizing the role of 1-heptyl-3-methylimidazolium IL in minimizing yellowing post-UV irradiation.

This research contributes to the broader field of paper conservation and cultural heritage preservation, offering practical insights into the application of ILs for restoring and maintaining paper-based artifacts. The results highlight the nuanced effects of different ILs on paper properties, providing useful information for conservators and researchers.

Future studies could delve deeper into the long-term effects of IL treatment on paper artifacts and explore additional IL formulations. The preservation of inked materials and a comprehensive understanding of ILs’ interaction with diverse inks warrant further investigation. Additionally, exploring the scalability and practical implementation of IL-based restoration processes would enhance the applicability of these findings in real-world conservation practices. This work paves the way for a nuanced understanding of ILs’ potential in cultural heritage preservation, opening avenues for sustainable and effective strategies in the field of paper restoration.

## Figures and Tables

**Table 1:** Specific consumption (S_c_) of paper restauration agents used in this study at the concentration of 10% *v*/*v* and the values supporting the deacidification of paper and changes in ash content.

Sample	S_c_ (g/g Paper)	Paper pH	Ash Content (%)	Paper Basis Weight (g/m^2^)
B1-PrEIMTs	0.17 ± 0.03	6.50 ± 0.20	0.93 ± 0.15	64 ± 0.25
B1-PentMIMTs	0.14 ± 0.01	6.70 ± 0.15	1.02 ± 0.18	65 ± 0.15
B1-HeptMIMTs	0.09 ± 0.01	7.00 ± 0.10	1.01 ± 0.15	65 ± 0.30

## Data Availability

The data presented in this study are available on request from the corresponding author.
